# Effect of tamsulosin on testis histopathology and serum hormones in adult rats: Experimental study

**DOI:** 10.18502/ijrm.v13i7.7370

**Published:** 2020-07-22

**Authors:** Yegane Kohestani, Bentolhoda Kohestani, Zahra Shirmohamadi, Masoumeh Faghani

**Affiliations:** ^1^Department of Anatomical Sciences, Bandar-Anzali Pardis, Faculty of Medicine, Guilan University of Medical Sciences, Bandar-Anzali, Iran.; ^2^Hematopoietic Stem Cell Research Center, Shahid Beheshti University of Medical Sciences, Tehran, Iran.; ^3^Department of Biostatistics, Shahid Beheshti University of Medical Sciences, Tehran, Iran.; ^4^Department of Anatomical Sciences, Faculty of Medicine, Guilan University of Medical Sciences, Rasht, Iran.

**Keywords:** Tamsulosin, Seminiferous tubules, Histopathology, Rat, Testis.

## Abstract

**Background:**

Tamsulosin is an inhibitory factor of alpha-adrenergic receptors that is used for relieving of the clinical symptoms and management of acute urinary retention.

**Objective:**

The aim of this study was to evaluate the effects of tamsulosin on the endocrine axis and testicular tissue in adult male rats.

**Materials and Methods:**

In this experimental study, 30 adult male Wistar rats (weighing 250-300 gr) were divided into three groups: 1) control (received distilled water), 2) experimental 1 (received 0.2 mg/kg/day tamsulosin) and 3) experimental 2 (received 0.4 mg/kg/day tamsulosin) through oral gavage for 28 days. Serum hormones level and testicular histopathology were evaluated at the end of the experiment.

**Results:**

In this study, the testicular weight decreased significantly in the experimental groups compared to the control group. A significant decrease was seen in testicular weight (p = 0.004) and the number of Leydig cells in tamsulosin-treated groups (p = 0.012). Tamsulosin improved the hormone profile in experimental groups. Also, higher dose of tamsulosin significantly changed the number of Leydig, spermatogonia cells, the thickness of germinal layer, and the diameter of the seminiferous tubules.

**Conclusion:**

Results showed that using tamsulosin, possibly reduces the testosterone concentration through adrenergic axis system and in turn has destructive effects on proliferative activity of germ cells.

## 1. Introduction

Benign prostatic hyperplasia (BPH) is considered as one of the most common diseases in men that can lead to lower urinary tract symptoms (LUTS). The prevalence of BPH significantly increases with age, so that, some studies show that the disease prevalence increases by up to 80% with aging (1, 2). Based on researches in Europe, United States, and Asia, old men have a higher risk factor for the onset and progression of the clinical BPH (1-5). In addition, prostate volume increases with age, so that according to Krimpen and Baltimore longitudinal studies on aging, the prostate hypertrophy rate is 2-2.5% annually (1, 6, 7). Therefore, prostate hypertrophy could be a risk factor for LUTS progression. The larger prostate is associated with benign prostatic enlargement (BPE). This enlargement increases the risk of BPH, urinary retention, and the need for prostate surgery (8).

LUTS are treated by alpha 1-adrenergic receptor blocking drugs and 5-alpha-reductase inhibitor (9, 10). Alpha1 receptors are predominantly present in the stromal tissue of prostate, prostatic urethra, and urinary bladder (11). In other words, the relaxation of the smooth muscles of the prostate and bladder is reduced LUTS in patients with BPH (11). In addition, it is reported that alpha-adrenergic receptor function depends on multiple effectors systems such as multiple G proteins (12). These proteins activate a series of cellular mechanisms that alter the levels of cyclic AMP and intra- and extra cellular Ca++ (13). Of note, Alpha adrenergic receptor agonist might activate these mechanisms (12).

Tamsulosin (Flomax) is considered as one of the selective antagonists for α1A and α1D adrenergic receptors. One of its side effects is disturbance in the male fertility system (13). There are several effective factors on male fertility reduction, including important congenital malformations of the genitourinary system, infectious diseases, genetic disorders, immunologic factors and endocrine axes disorders, painful or difficult urination, abnormal ejaculation, and back pain (14). However, idiopathic cause was found in 60-70%of cases in male infertility (15).

Besides, most researchers, on the other hand, have investigated the effect of tamsulosin in the treatment of BPH (16). Researchers have reported that the use of tamsulosin relaxes the prostate and bladder neck muscles and increases bladder emptying (17). They have focused on tamsulosin therapeutic effects that reduce urinary retention and prostate hyperplasia; however, the probable cytotoxic effects of it on testicular tissue have not been considered yet. It is worth nothing that increasing old population in most societies resulted in higher incidence of BPH and LUTS. Therefore, to reduce the symptoms of patients with BPH and LUTS, tamsulosin is considered as a safe drug (16, 18) and is prescribed routinely; however, its effect on endocrine axis and histology of testicular tissue has not been fully studied. Thus, the present study was aimed to evaluate the effect of tamsulosin on serum concentration of male hormones and histopathology of the testicular tissue.

## 2. Materials and Methods

### Animals and treatment

Thirty adult male Wistar rats (weighing 250-300 gr) were purchased from the Pasteur Institute of Iran. Rats were kept under standard conditions for one week for adaption to new environment (temperature of 21 ± 2°C and 12 hr light/darkcycle). The control group (N = 10) received distilled water (solvent for tamsulosin), the experimental group 1 received 0.2 mg/kg/day tamsulosin, and the experimental group 2 received 0.4 mg/kg/day tamsulosin based on human dosage (19, 20). Animals were gavaged with 1 ml distilled water or tamsulosin solution for 28 consecutive days (19).

### Blood sampling and testis weight measurement

After 28 days, the rats were anesthetized and then sacrificed with diethyl ether. The blood samples were obtained directly from their hearts, and then the left testis was removed and weighed.

### Preparing the tissue samples

In order to examine the testis histological changes, the left testis was fixed for seven days in a Modified Davidsons Fluid (MDF) solution containing 30% formaldehyde, 15% ethanol, 5% glacial acetic acid, and 50% distilled water. The testes were then washed with PBS solution and cut into smaller pieces. The MDF-fixed testes were dehydrated and embedded in paraffin.

Sections measuring 5 µm were prepared and at least five slides from each testis were stained with Heiden Hain Azan for histopathological evaluation. This was performed according to the routine tissue-staining methods. In order to evaluate the testis tissue histopathological changes, four slices were assessed from each mouse. Five microscopic fields from each slide were observed at 400× magnification using a standard light microscope (Olympus, Japan). Spermatogonia, Sertoli, and Leydig cells were counted using the light microscope, and the images were prepared by a digital camera (BX 51 Japan) calibrated with a hemocytometer slide share attached to the microscope. Germinal epithelium area, seminiferous tubules diameter, and number of seminiferous tubules and interstitial cells were measured using the Image J 1.44P software (National institute of Health, USA).

### Serum hormones level analysis

After anesthesia, the rats' blood samples were immediately obtained directly from their heart to determine the serum level of testosterone, LH, and FSH at the end of the study. Then, the blood samples were centrifuged at 3000 rpm for 20 min. Hormones level was measured using the Testosterone ELISA kits (Abcam, ab108666), EKU05694 Rat Luteinizing Hormone (LH) ELISA kit (ZellBio Germany), and EKU04249 Rat Follicle Stimulating Hormone (FSH) ELISA kit (ZellBio Germany). All reagents, standards, and samples were prepared according to the instructions presented in the kit.

### Ethical consideration

Animals were maintained in accordance with the ethical standards approved by the Animal Care Committee of Guilin University of Medical Sciences (IR.GUMS.REC.1398.388).

### Statistical analysis

The results were presented with mean and standard deviation. Data were analyzed using the Statistical Package for the Social Sciences, version 18.0, SPSS Inc, Chicago, Illinois, USA (SPSS), and p < 0.05 was considered as significant. ANOVA and Tukey's test were used to compare the parametric parameters (testicular weight, testosterone, LH and FSH concentrations, and number of spermatogonia, Leydig, and Sertoli cells). Non-parametric test (Kruskal-Wallis H) was used to compare the histopathological grades (diameter of cells and the area of germinal epithelium) and the non-parametric test (Mann-whitney U) with modification of pairwise comparisons (to control the first type error) was used if the results test was significant. Kolmogorov-smirnov test was used to determine the normal distribution of data.

## 3. Results

A statistically significant difference was detected between the testes weight in all groups (p = 0.004), but there were no significant differences between the control and experimental groups 1 (p = 0.98) (Table I). However, significant differences were observed in the testes weight of the control and experimental group 2 (p = 0.008) and the experimental groups 1 and 2 as well (p = 0.012).

**Figure 1:**
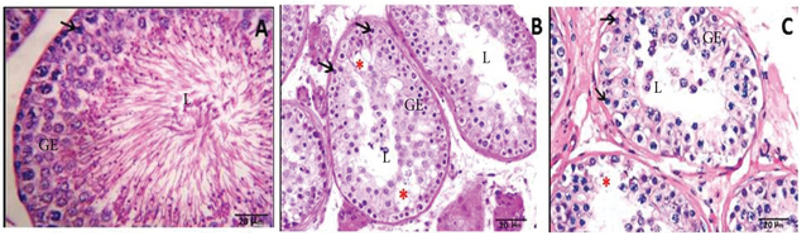
Microscopic image of testicular tissue sections in different groups (at 400x magnification; scale bar: 20 microns; and staining method: Heiden Hain Azan). (A) Control group (the group that received distilled water): The testicle tissue is normal in terms of the number of spermatogonia, the thickness of reproductive epithelium, and the diameter of the seminiferous tubules. (B) Experimental group 1 (the group that received 0.2 mg/kg/day tamsulosin): According to the testicular tissue in this group, spermatogonia, reproductive epithelial thickness, and the number of spermatogonia reduced. (C) Experimental group 2 (the group that received 0.4 mg/kg/day tamsulosin): The testicular tissue in this group shows a very severe reduction in the thickness of reproductive epithelium and its degeneration and a significant decrease in spermatogonia.

Our findings revealed that tamsulosin significantly decreased the plasma concentration of testosterone in comparison with the control (p = 0.0001) (Table I). Moreover, testosterone level decreased more in the experimental group 2 compared to the experimental group 1 (p = 0001) (Table I; Figure 1). Of note, tamsulosin significantly increased the FSH level in the experimental groups (p = 0.009).

The spermatogenesis was evaluated in germinal epithelium through spermatogonia cell numbers. It showed a significant decrease in the numbers of spermatogonia across all groups (p = 0.0001). In addition, there was a significant difference in the number of spermatogonia between the experimental groups in dose-dependent manner of tamsulosin (Table II). Furthermore, areas of reproductive epithelium remarkably reduced in the experimental groups (p = 0.0001). Interestingly, some vacuoles were observed in germinal epithelium especially in the experimental group 2 (Figure 1).

**Table 1:** Comparison of testes weight and serum testosterone concentrations in the control group and the groups treated by tamsulosin (n = 10)


**Parameters** **Groups**	**Control (mean ± SD)**	**Experimental 1 (mean ± SD)**	**Experimental 2 (mean ± SD)**	**P-value**
**Testicle weight (gr)**	1.00 ± 0.15	0.99 ± 0.15${}^{b}$	0.80 ± 0.10${}^{a}$	0.004
**Testosterone (ng/ml)**	1.05 ± 0.21	0.81 ± 0.09${}^{c}$	0.60 ± 0.10${}^{b}$	0.0001
**LH (ng/ml)**	0.194 ± 0.048	0.172 ± 0.051	0.155 ± 0.039	0.186
**FSH (ng/ ml)**	0.202 ± 0.048	0.254 ± 0.042	0.304 ± 0.057${}^{a}$	0.0001
Mean ± SD, one-way ANOVA, and post-hoc tests;${}^{a}$Significant between the control group and the experimental group 2; ${}^{b}$Significant between the experimental groups 1 and 2; ${}^{c}$Significant between the control group and the experimental group 1; Control: The group that received distilled water; Experimental 1: The group that received 0.2 mg/kg/day tamsulosin; and Experimental 2: The group that received 0.4 mg/ kg/day tamsulosin. LH: Luteinizing hormone; FSH: Follicle-stimulating hormone; SD: standard deviation				

**Table 2:** Comparison of number of spermatogonia, area of reproductive epithelium, and seminiferous tubules diameter in the different groups (n = 10)


**Parameters** **Groups**	**Control (mean ± SD)**	**Experimental 1 (mean ± SD)**	**Experimental 2 (mean ± SD)**	**P-value**
**Spermatogonia number**	37.52 ± 4.14	32.43 ± 6.06${}^{a}$	23.03 ± 4.88${}^{b}$	0.001
**Area of reproductive epithelium**	36661.65 ± 5706.4	30009.12 ± 8005.49${}^{a}$	15344.34 ± 4164.54${}^{b}$	0.001
**Seminiferous tubules diameter**	51498.95 ± 6597.87	45517.13 ± 6214.75${}^{a}$	20983.23 ± 6679.39${}^{b}$	0.001
**Sertoli cell number**	17.168 ± 4.060	16.588 ± 4.543	15.153 ± 5.048	0.603
**Leydig cell number**	16.513 ± 5.112	14.193 ± 3.701	11.165 ± 1.072${}^{c}$	0.012
Mean ± SD, one-way ANOVA, and post-hoc tests,${}^{a}$Significant between the control group and the experimental group 1(0.2 mg/kg/day);${}^{b}$Significant between the experimental groups 1 and 2;${}^{c}$Significant between the control group and the experimental group 2; Control: The group that received distilled water; Experimental 1: The group that received 0.2 mg/kg/day tamsulosin; and Experimental 2: The group that received 0.4 mg/kg/day tamsulosin. SD: standard deviation				

## 4. Discussion

The present study showed that tamsulosin resulted in pathological changes in testis and the secretion of testosterone in male rats. In addition, it is suggested that tamsulosin could inhibit androgenesis process. The serum testosterone level was decreased significantly among the experimental groups (p < 0.05), which indicates the strong impact of higher dose of tamsulosin on androgenesis. It is suggested that the tamsulosin exerts its function on the activity of gonadotropin-releasing hormone under the influence of norepinephrine by alpha-adrenergic receptors. Norepinephrine, as a neurotransmitter that is secreted from adrenergic neurons, has a significant impact on adrenergic receptors, especially alpha receptors. Also, these adrenergic neurons can target gonadotropin-releasing hormone (14, 21, 22). Furthermore, norepinephrine can release the LH-releasing hormone.

According to Selvage's findings, prazosin and adrenergic receptors inhibitor reduced the production of LH-releasing hormone in the hypothalamus and resulted in LH decrease (23). In other words, this axis is able to influence the secretion of testosterone through the inhibition of LH. In addition, some studies have shown that the alternative releasing of LHRH is modulated by adrenergic neurons, particularly norepinephrine, via alpha1-adrenergic receptors (22, 24).

Alpha-blockers are usually used as the first choice of treatment for patients suffering from LUTS because the safety and effectiveness of these have been confirmed in many randomized studies (17, 25, 26).

Androgens exert their effects on prostate proliferation by binding and activating the androgen receptors. Anti-androgens competitively inhibit the binding of the receptors by agonists. When anti-androgens are combined to their receptors, they, therefore, don't bind to DNA and transcription would be inhibited (27).

On the other hand, based on Gotkas and coworker's study, the advantage of taking single dose of tamsulosin compared to the rest of α1-adrenoceptor antagonist is not convincing enough for this medicine to be used in all patients (20). Therefore, it seems that clinicians routinely use different doses of tamsulosin depending on the severity of the disease. In addition, α-blockers often influence the secretion of testosterone. According to our results, taking 0.2 and 0.4 mg/kg of tamsulosin for 28 days significantly decreased the testosterone concentration and disturbed the testicular tissue histology in the experimental groups. This may be described by the harmful effects of tamsulosin on the gonadotropin-hypothalamic axis and Leydig cells. Our finding was confirmed by the reduction of the numbers of Leydig cells between the experimental groups (p = 0.009).

Some studies used 0.4 mg/kg of tamsulosin to treat BPH based on the severity of the disease and did not consider the side effects of tamsulosin in reducing spermatogonia and Leydig cells (26, 28). Of note, our study showed that tamsulosin at a dose of 0.2 mg/kg can also significantly reduce the number of spermatogonia. and Leydig cells. Significant reduction of androgens with a 0.2 mg/kg of tamsulosin can sufficiently reduce androgens for the desirable therapeutic effect. To prevent the side effects of tamsulosin, it is recommended to use a lower dose of 0.2 mg/kg for the treatment of LUTS.

Our study revealed a significant decrease in the number of spermatogonia, the areas of reproductive epithelium, and the diameter of seminiferous tubules in the experimental groups (p < 0.001), which indicates the destructive effect of tamsulosin in the testicular tissue. In addition, our study showed that there were many vacuoles in germinal epithelium in experimental groups 1 and 2 compared to the control group that may indicate tissue damage in the germinal epithelium. Our data are consistent with the results of previous studies on the dose-dependent use of this drug. Beltagy and coworkers found that the treatment with tamsulosin hydrochloride had more harmful side effects than finasteride on rats, which leads to the disturbances in prostatic, testicular function, and neurotransmitters indices (29). Besides, they also reported that combined treatment of finasteride with tamsulosin hydrochloride has lower side effects than tamsulosin hydrochloride alone.

In another study, researchers combined the tamsulosin with non- α-blockers such as Omega 3 fatty acid and the results were fantastic. They reported that this co-treatment had better clinical results (30). In another study, drugs combination therapy was used to reduce the deleterious effects of tamsulosin. They showed that the combination of three drugs LDD175, tamsulosin, and finasteride is more effective in the treatment of BPH patients because some patients are ressistnt to individual administration of these drugs (31). On In another study, the researchers observed a significant decrease in the number, mobility, activity, and survival of the epididymal sperm after tamsulosin injection, especially at higher doses. Higher doses of drug results in a significant decrease in testosterone levels, testes and body weight, diameter of seminiferous tubules, and number of Leydig cells (32). It seems that tamsulosin has destructive effects on testis tissue, imbalance hormones level and infertility. Therefore, using low dose of this drug alongside the application of other proper antagonist drugs is recommended.

## 5. Conclusion

Finally, according to the results of this study, the secretion of testosterone hormone reduced after the administration of tamsulosin and could possibly have a significant effect on hypothalamic- hypophysial axis. Testosterone impacts the activity of the gonadotrophin-releasing hormone, which is itself influenced by norepinephrine and adrenergic receptors. Considering the AUA Guideline and ejaculatory dysfunction at higher doses of tamsulosin, it is better to avoid using higher doses of tamsulosin to prevent its adverse side effects on testicular tissue. It is revealed that histopathological changes of testicular tissue resulted in the reduction of hormone levels. Therefore, it is suggested that a combination therapy with other drugs could probably reduce the harmful side effects of tamsulosin.

##  Conflict of Interest

The authors declare no conflict of interest.
